# Non-Avian Animal Reservoirs Present a Source of Influenza A PB1-F2 Proteins with Novel Virulence-Enhancing Markers

**DOI:** 10.1371/journal.pone.0111603

**Published:** 2014-11-04

**Authors:** Irina V. Alymova, Ian A. York, Jonathan A. McCullers

**Affiliations:** 1 Influenza Division, National Center for Immunization & Respiratory Diseases, Centers for Disease Control & Prevention, Atlanta, Georgia, United States of America; 2 Department of Infectious Diseases, St. Jude Children's Research Hospital, Memphis, Tennessee, United States of America; 3 Department of Pediatrics, University of Tennessee Health Science Center, Memphis, Tennessee, United States of America; The University of Chicago, United States of America

## Abstract

PB1-F2 protein, expressed from an alternative reading frame of most influenza A virus (IAV) PB1 segments, may possess specific residues associated with enhanced inflammation (L62, R75, R79, and L82) and cytotoxicity (I68, L69, and V70). These residues were shown to increase the pathogenicity of primary viral and secondary bacterial infections in a mouse model. In contrast to human seasonal influenza strains, virulence-associated residues are present in PB1-F2 proteins from pandemic H1N1 1918, H2N2 1957, and H3N2 1968, and highly pathogenic H5N1 strains, suggesting their contribution to viruses' pathogenic phenotypes. Non-human influenza strains may act as donors of virulent PB1-F2 proteins. Previously, avian influenza strains were identified as a potential source of inflammatory, but not cytotoxic, PB1-F2 residues. Here, we analyze the frequency of virulence-associated residues in PB1-F2 sequences from IAVs circulating in mammalian species in close contact with humans: pigs, horses, and dogs. All four inflammatory residues were found in PB1-F2 proteins from these viruses. Among cytotoxic residues, I68 was the most common and was especially prevalent in equine and canine IAVs. Historically, PB1-F2 from equine (about 75%) and canine (about 20%) IAVs were most likely to have combinations of the highest numbers of residues associated with inflammation and cytotoxicity, compared to about 7% of swine IAVs. Our analyses show that, in addition to birds, pigs, horses, and dogs are potentially important sources of pathogenic PB1-F2 variants. There is a need for surveillance of IAVs with genetic markers of virulence that may be emerging from these reservoirs in order to improve pandemic preparedness and response.

## Introduction

Influenza A viruses (IAVs) are endemic in several species of domestic poultry and mammals. These viruses form a vast reservoir from which both intact viruses, and individual virus gene segments, occasionally emerge to affect humans. It is therefore important to understand the extent of the public threat posed by influenza viruses circulating in domestic animals.

Birds, especially waterfowl, are the natural reservoirs from which most IAVs have emerged, and almost all known subtypes of IAVs can infect birds [1]. Avian IAVs of the H5N1, H7N7, H7N9, and H9N2 subtypes circulate in domestic poultry and have sporadically crossed over into humans and caused illness [2]–[5]. However, these viruses have not yet achieved sustained human-to-human transmission. In contrast, avian viruses donated genome segments during reassortment with endemic human viruses, resulting in H2N2 and H3N2 IAV human pandemics in 1957 and 1968, respectively [6], [7].

IAV are also endemic in a number of non-human mammals, including horses, dogs, and pigs. The close contact between humans and domestic animals means that IAV of these species may also pose a pandemic threat, either directly or via reassortment. In particular, swine IAVs were the source of the most recent human IAV pandemic, the H1N1pdm09 virus of 2009 [8], and some evidence suggests that the 1918 H1N1 pandemic, which originated from an avian IAV, may also have had a swine as an intermediate host [9]. Virologic surveillance programs currently conducted by public and animal health authorities have mainly focused on changes in the genes encoding two IAV surface glycoproteins, hemagglutinin and neuraminidase. However, other genes are also associated with influenza virus virulence [1]. Among other viral factors, the IAV PB1-F2 protein [10] was found to be capable of greatly enhancing lung inflammation and promoting secondary bacterial pneumonia (which is one of the major causes of excess mortality observed during influenza pandemics) in a mouse model [11], [12].

PB1-F2 is a small protein (up to 90 amino acid long) encoded in the second reading frame of the PB1 gene segment [10], that contributes to IAV pathogenesis in several ways. We recently identified four amino acid residues (L62, R75, R79, and L82, according to PB1-F2 numbering; hereafter referred to as inflammatory residues) in the C-terminal part of the protein, that enhance inflammation in mouse lungs, resulting in acute lung injury [13]. We also identified three residues—I68, L69, and V70 (hereafter referred to as cytotoxic residues)—that enhance cell cytotoxicity [14]. The presence of either the inflammatory or cytotoxic residues (hereafter referred to as virulent residues) in PB1-F2 significantly promoted the development of secondary bacterial pneumonia in a mouse model.

All pandemic H1N1 1918, H2N2 1957, and H3N2 1968, and most highly pathogenic H5N1 IAVs maintained full-length pathogenic (e.g. causing excessive lung inflammation and/or promotion of secondary bacterial infection) PB1-F2 phenotype and contained all 4 inflammatory residues, which may have contributed to excessive lung inflammation and promotion of secondary bacterial pneumonia. During adaptation into human seasonal strains the virulent PB1-F2 phenotype was diminished either through truncation (in the H1N1 lineage) or mutations (in the H3N2 lineage). It was proposed that the inflammatory (L62, R75, R79, and L82) and cytotoxic (I68, L69, and V70) residues are PB1-F2 genetic markers of virulence and suggested surveillance for these residues in human and animal isolates expressing the full-length PB1-F2 protein [15], [16].

Previous sequence analysis of avian influenza viruses (AIV) PB1-F2 proteins of the H5N1 lineage (including those from highly pathogenic viruses) showed that 610 of 766 sequences (79.6%) available in the National Center for Biotechnology Information (NCBI) Influenza Virus Research Database encode PB1-F2 proteins with all 4 inflammatory residues [17]. In addition, most recently emerged zoonotic IAVs of avian H7N7, H7N9, and H9N2 lineages express full-length PB1-F2 proteins with inflammatory markers (unpublished data).

In this study, we determined the occurrence and distribution of PB1-F2 proteins with virulent residues in non-avian animal reservoirs such as pigs, horses, and dogs, with the goal of understanding the potential for these domestic animals to donate virulent PB1-F2 to humans either by zoonotic infection, or through reassortment with endemic human viruses.

## Methods

### Analysis of influenza A virus PB1 proteins

**Figure 1:**
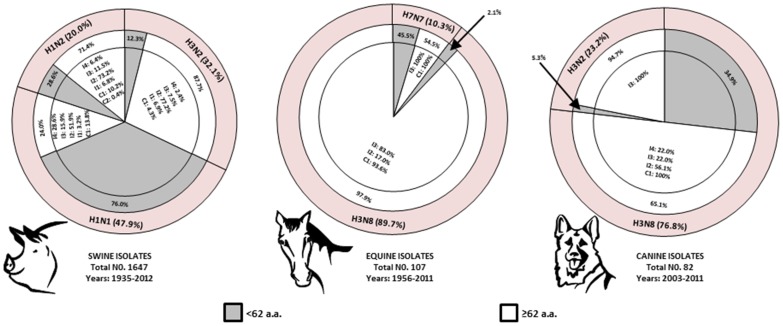
Genetic markers of virulence in the PB1-F2 proteins from influenza viruses of domestic mammals. The IAV isolates of swine, horses, and dogs were categorized into the lineage (outer ring), PB1-F2 length (middle ring), and combination of inflammatory (indicated as “I”) and cytotoxic (indicated as “C”) residues (inner ring). Within the outer ring, the percentage of each grouping is shown relative to the total number of isolates. Within the middle and inner rings, the percentage of each grouping is shown relative to the preceding grouping. The numbers of inflammatory (indicated as “I1” through “I4”) and cytotoxic (indicated as “C1” and “C2”) residues were determined for PB1-F2 proteins with length of 62 or more amino acids (e.g. capable of encoding either inflammatory or cytotoxic residues).

**Figure 2:**
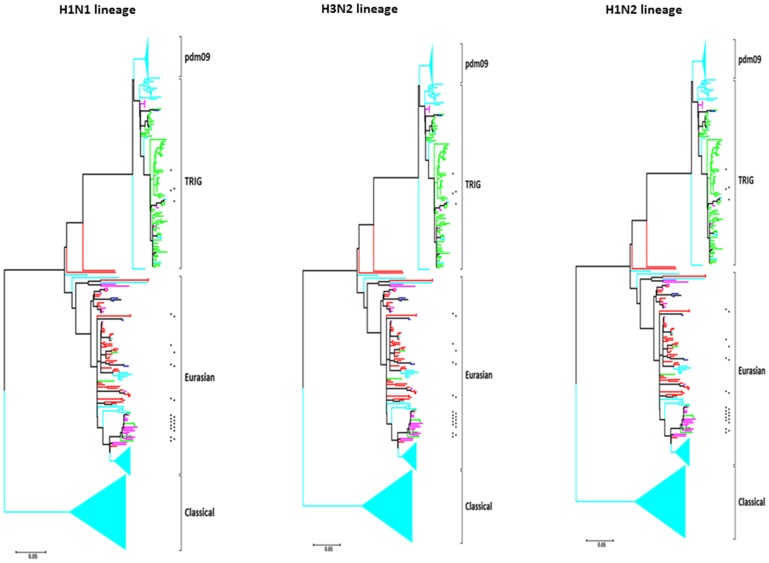
Phylogeny overview of swine influenza A PB1 gene. Phylogenetic analysis for 789 H1N1, 529 H3N2, and 329 H1N2 SIVs are shown. 1, 2, 3, and 4 inflammatory residues are indicated in blue, green, fuchsia, and red, respectively. The presence of a cytotoxic residue is indicated by an asterisk. Cyan indicates PB1-F2 proteins truncated before residue 62. More detail, including strain names and bootstrap values, is shown in Figure S1.

**Figure 3:**
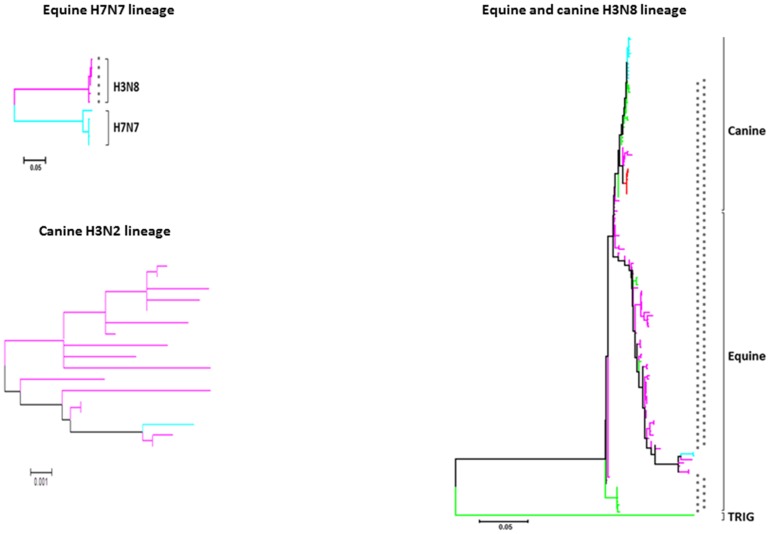
Phylogeny overview of equine and canine influenza A PB1 genes. Maximum likelihood phylogenetic trees for 96 equine and 63 canine H3N8, 11 equine H7N7, and 19 canine H3N2 viruses were generated. 1, 2, 3, and 4 inflammatory residues are indicated in blue, green, fuchsia, and red, respectively. The presence of one or two cytotoxic residues is indicated by one or two asterisks, respectively. Cyan indicates PB1-F2 protein truncated before residue 62. More detail, including strain names and bootstrap values, is shown in Figure S2.

Nucleotide sequences of PB1 proteins of well-established swine (H1N1, H1N2, and H3N2), equine (H7N7 and H3N8), and canine (H3N2 and H3N8) lineages existing in the NCBI (http://www.ncbi.nlm.nih.gov/genomes/FLU/FLU.html) and GISAID (http://platform.gisaid.org; see Table S1 for acknowledgements) influenza virus resource databases were accessed on January 20, 2013. Rare viruses of other subtypes (e.g., H5N1 in swine or canine) were not included, as they generally represent cross-infection with avian viruses that do not persist and evolve in the new host. After removing duplicate strains and partial PB1 sequences that did not contain the PB1-F2 region, we analyzed 1647 swine (isolated from 1935–2012), 107 equine (1956–2011), and 82 canine (2003–2011) influenza viruses. The PB1-F2 regions from these sequences were translated and analyzed for the presence of the inflammatory (L62, R75, R79, and L82) and cytotoxic (I68, L79, and V70) residues (Figure 1). Next, sequences covering full-length PB1 were aligned using MAFFT [18] (excluding PB1 sequences that were identical at the nucleotide level to a sequence already included in the alignment), and phylogenetic trees (shown as an overview in figures 2 and 3, and as full text in Figures S1 and S2) were generated. Unrooted phylogenetic trees were constructed using maximum likelihood analysis with the nearest-neighbor-interchange method, based on the general time reversible model with gamma distributed rate heterogeneity with invariant site (G+I), implemented in MEGA 5.1 [19]. The reliability of the internal branches was evaluated using 500 bootstrap replicates; nodes supported at ≥80% are indicated in Figures S1 and S2.

## Results and Discussion

### Distribution of PB1-F2 virulence markers in swine influenza viruses

Multiple lineages of swine influenza viruses (SIVs) circulate in domestic pigs [20]. Classic SIVs, which arose from the H1N1 human pandemic strain of 1918, quickly entered the swine population and remained largely unchanged for more than 75 years. Since the late 1990s, however, swine influenza has developed from a seasonal disease caused by a single fairly stable H1N1 genotype to an endemic year-round respiratory infection triggered by multiple genetically unstable H1N1, H1N2, and H3N2 SIV lineages. Infection of pigs with H9N2, H5N1, H3N8, H2N1, H3N1, H4N1, and H2N3 subtypes has also been documented [20]. Epizootic swine influenza is characterized by acute onset of respiratory disease with high morbidity but low mortality.

Of the 1647 swine isolates in our analysis, 47.9% were H1N1, 32.1% were H3N2, and 20.0% were H1N2 subtypes (Figure 1). More than 70% of H3N2 and H1N2 SIVs encode a PB1-F2 at least 62 residues long and are therefore able to encode at least one virulent residue. In contrast, about 76% (600 of 789 isolates) of H1N1 SIV PB1-F2 proteins were truncated before residue 62 and cannot encode either inflammatory or cytotoxic residues. Considering only the PB1-F2 proteins of at least 62 residues, the majority of SIV PB1-F2 proteins from all of the three predominant lineages had a combination of two inflammatory residues (77.2% for H3N2, 73.2% for H1N2, and 51.9% for H1N1). The combination of all 4 (L62, R75, R79, and L82) or any 3 inflammatory residues was rare in H1N2 and H3N2 SIV lineages (found in 2.4% to 11.5% of isolates). Those viruses in the H1N1 lineage of SIVs in which PB1-F2 encoded the region of virulent residues (amino acids 62 through 82) had substantial fractions of PB1-F2 proteins with either all 4 (28.6%) or any 3 (15.9%) inflammatory residues. A single cytotoxic residue was present in only 4.3% to 13.7% of SIV PB1-F2 proteins (Figure 1). The most common cytotoxic residue in SIVs was I68 (which induces the most prominent pathogenic effect in mice among the 3 cytotoxic residues; unpublished data), although a few V70 residues were also observed. Of note, the presence of cytotoxic residues was always associated with inflammatory markers in SIVs.

SIV evolution is complex and involves repeated introduction of avian influenza virus genes and multiple reassortment events, so that PB1 genes from multiple origins are distributed across the various HA and NA subtypes. Considering PB1 lineages separately from HA and NA subtypes helps clarify the evolution of the virulent PB1-F2 residues. Virtually all the PB1-F2 that originated from the “classical” (a descendant of the H1N1 1918 human pandemic influenza), “Asian” (with PB1 from human H1N1), and “H1N1pdm09” (with PB1 from human H3N2 via the swine triple-reassortant internal gene [TRIG] cassette) origin PB1 lineages were truncated before residue 62 (Figures 2 and S1). Interestingly, in contrast to PB1-F2 of “classical” SIVs, the PB1-F2 from its ancestor, the human 1918 pandemic H1N1, was full-length and contained all 4 inflammatory residues. Truncated PB1-F2 proteins were seen more sporadically in other lineages. Thus, PB1-F2 from the Eurasian lineage (an avian-origin strain) frequently had 3 or 4 inflammatory residues and frequently contained the I68 cytotoxic residue. The TRIG cassette lineage (with the PB1 of human H3N2 origin) predominately had 2 inflammatory residues, L62 and L82 but only rarely and sporadically had cytotoxic residues.

The human seasonal H3N2 lineage (repeatedly isolated from Asian swine between 1997 and 2005) did not contain any inflammatory residues and, on its initial entry into the swine population, did not contain cytotoxic residues. However, between 2000 and 2004, a small cluster of these viruses did acquire a cytotoxic residue (either I68 or V70).

### Distribution of virulent PB1-F2 proteins in equine and canine influenza viruses

Equine influenza is a major cause of respiratory disease in horses and, despite the availability of vaccines, continues to cause problems around the world. The predominant equine influenza virus (EIV) subtype today, H3N8, was introduced into horses from aquatic birds as early as 1963 [21]. A formerly common equine influenza lineage, H7N7, is now extinct. Of the 107 equine isolates in our analysis, 89.7% were H3N8 and 10.3% were H7N7 lineages (Figure 1). About 98% of H3N8 and 54.5% of H7N7 EIV PB2-F1 genes encoded proteins that were at least 62 residues in length, long enough to contain the full or partially truncated C-terminus. Most of the viruses from both lineages (100% of H7N7 and 83.0% of H3N8) had a combination of any 3 inflammatory residues. The remaining 17.0% of H3N8 EIVs contained a combination of two inflammatory residues (L62 with either R75 or L82 amino acids; not shown).

At least two lineages of IAV, equine-origin H3N8 [22] and avian-origin H3N2 [23], have adapted to canine populations and are circulating in America, Europe, and Asia. Although H3N8 canine influenza virus (CIV) is usually a mild disease, some dogs may develop severe pneumonia, resulting in death [22]. Infection of dogs with H3N2 CIV is often associated with severe respiratory disease and high mortality [24]. Dogs are also occasionally susceptible to natural influenza infections from avian H5N1 or human pandemic H1N1/2009 and H3N2 IAVs [25], although there is no evidence of sustained transmission. Of the 82 canine isolates we analyzed, 76.8% were H3N8 (derived from EIV) and 23.2% were H3N2 (derived from AIV) subtypes (Figure 1). Analysis of the C-terminal ends showed that 94.7% of H3N2 and 50.8% H3N8 CIVs are long enough to encode a region of virulent residues. While none of the H3N2 lineage PB1-F2 had all 4 inflammatory residues, aproximately 22.0% of those from the H3N8 lineage of CIVs had all 4 residues. Interestingly, despite the fact the dogs acquired the H3N8 lineage from horses, none of H3N8 EIVs had all 4 residues in PB1-F2 (Figure 1). This observation confirms the proposition that CIV is now undergoing selection in dogs that is leading to distinct CIV and EIV lineages [26]. The combination of any 3 inflammatory residues was present in all H3N2 and 22.0% of H3N8 CIVs. The grouping of L62 and R75 was typical for the canine host and was found in 56.0% of H3N8 CIVs (not shown).

Cytotoxic residues were common in EIV (93.6% of H3N8, 100% of H7N7 lineages) and CIV (100% of H3N8 lineage PB1-F2 of at least 62 residues) (Figure 1). Similar to SIVs, I68 (always in combination with inflammatory residues) was present in equine and canine isolates. In only one case was more than one cytotoxic residue present (H3N2 A/canine/Colorado/30604/2006, which contains I68 and V70 amino acids; not shown). The combination of inflammatory and cytotoxic residues was also observed in some PB1-F2 proteins from human IAVs, such as H1N1 1918 pandemic PB1-F2 [12] and A/Taiwan/3355/97, a virus isolated from a patient with severe pneumonia [27]. Further studies will determine whether the presence of cytotoxic and inflammatory residues in PB1-F2 protein synergistically enhances primary viral and secondary bacterial infections.

Phylogenetic analysis of PB1 genes from EIVs indicated that all viruses of H7N7 lineage circulating until 1973 had PB1-F2 truncated before residue 62 (Figures 3 and S2). In the 1960s or 1970s, the original H7N7 PB1 segment was replaced by PB1 from the co-circulating avian-origin H3N8 lineage [28], which contained with full length PB1-F2 and 2 or 3 inflammatory and 1 (I68) cytotoxic residues.

In contrast to the SIVs, evolution of equine and canine IAVs of the H3N8 subtype is relatively straightforward, with little inter-subtype reassortment (Figures 3 and S2; [29]). For approximately 30 years after establishment of avian H3N8 in the equine population, PB1-F2 maintained its full length. From 1975 on, all the EIV H3N8 PB1-F2 proteins that were longer than 62 residues contained I68, as did all the CIVs that were derived from this lineage. In the early 2000s, EIV PB1-F2 became truncated before residue 82, though still maintaining 3 inflammatory residues.

This pattern of shortened PB1-F2 containing 3 inflammatory residues was seen in the earliest canine isolates from 2003, at about the time of introduction of equine H3N8 into dogs (Figures 3 and S2). In 2006–2007, a cluster of viruses, isolated from Florida, Pennsylvania, and Kentucky, restored a region of virulent residues in the PB1-F2 sequence, adding the fourth inflammatory residue (L82) in the process. However, after 2007, CIVs have had first a 79-residue protein containing 2 inflammatory residues, and then, after 2009, a PB1-F2 protein truncated before residue 62. This suggests that CIV containing full-length PB1-F2 may be less well adapted to dogs (probably due to interactions with unknown host factors) and, that during passage of the H3N8 lineage in dogs the virus is adapting to this species by eliminating virulent PB1-F2 residues, mainly through truncation rather than mutation. In principle, this may be similar to the H1N1 lineage of human IAV, which predominately has truncated PB1-F2, rather than to human H3N2 IAV, in which virulent PB1-F2 residues have been lost mainly through mutation.

In contrast to H3N8, all avian-origin H3N2 CIVs had 3 inflammatory and no cytotoxic residues, except for one virus (H3N2 A/Canine/Guangdong/2/2006) in which PB1-F2 is truncated at residue 57 (not shown).

Our phylogenetic analysis suggests that after entering the swine, equine, and canine populations, avian PB1-F2 rapidly undergoes a series of sequence changes. As avian influenza viruses adapt to mammals, there is a trend toward a reduction in the number of virulent residues over time, due to either PB1-F2 point mutations or, more commonly, truncation (Figures 2 and 3). These observations suggest that the IAVs containing virulent residues (particularly inflammatory residues) may be less well adapted to mammalian hosts and that evolution either of truncated PB1-F2 or of PB1-F2 variants that are less inflammatory, may contribute to virus fitness and transmission. One possibility is that such variants may persist better in individual hosts due to reduced inflammation, thus both extending the window of virus transmission and reducing disease severity. However, viruses containing inflammatory and cytotoxic residues can still efficiently infect and transmit between mammalian hosts, as evidenced by many of the Eurasian SIV lineage, EIV, and CIV, as well as human pandemic H1N1 1918 and H3N2 1968 IAVs. A virus capable of efficient human-to-human transmission, but containing virulent residues in PB1-F2, could therefore cause widespread, severe disease. The present analysis shows that such residues remain common among swine, equine, and canine influenza viruses (Figures 1 and 2). Thus, these hosts should be considered potential candidates for transmission of IAVs with virulent PB1-F2 proteins into the human population.

Similar to what is seen in humans, a combination of viral, bacterial, and host factors seems to contribute to the severity of primary influenza and secondary bacterial infections in swine, equine, and canine hosts. To date, there have been only two publications reporting the ability of SIV PB1-F2 proteins from H1N1 and H3N2 lineages to modulate virus replication, lung histopathology, and innate immune responses in pigs, and this ability appears to be strain-dependent [30], [31]. The role of IAV PB1-F2 proteins in the severity of canine and equine influenza-associated respiratory disease has not been explored. Interestingly, some EIV strains were reported to be more pathogenic than others. Differences in pathogenicity between EIVs are linked to the induction of different levels of such pro-inflammatory cytokines as IL-6 and IFN-α [32], [33]. Studies in horses have focused on the role of the NS1 protein (which interferes with the induction of type 1 interferon [34]) in cytokine production. However, virulent variants of PB1-F2 proteins with inflammatory and cytotoxic residues also have potential to contribute to this phenomenon. Recent studies with H3N2 CIV, which has 3 inflammatory residues in PB1-F2, showed that severe morbidity and enhanced mortality observed in dogs infected with this lineage is due to induction of genes related to inflammation and apoptosis [24]. This observation can be correlated with the transcriptomic analysis of host immune and cell death responses after infection with mouse-adapted H1N1 A/WSN/33 (which has virulence-associated residues in its PB1-F2) showed an increase in the number and level of expression of activated genes linked to cell death, inflammation, and neutrophil chemotaxis due to PB1-F2 expression [35].

The severity of influenza-associated respiratory disease significantly increases if it is complicated by secondary bacterial infections. Pigs, horses, and dogs are also highly susceptible to secondary bacterial infections with influenza. Secondary bacterial infections are the most common complicating factors in SIV outbreaks [26], and in horses, influenza infection aggravated by secondary bacterial infections may produce serious disease, such as pneumonia and pleuropneumonia [36]. Secondary bacterial pneumonia appears to have contributed significantly to deaths in dogs during the 2007 H3N2 IAV outbreak [23]. Enhancement of lung inflammation by virulent SIV, EIV, and CIV PB1-F2 proteins with L62, R75, R79, and L82 residues could contribute to the development of secondary bacterial infections in those cases. Infection of mice with naturally occurring SIVs with the highest number of inflammatory residues (L62, R75, R79, and L82) in PB1-F2 induced the highest incidence of death due to secondary bacterial pneumonia (about 90%), far worse than the rate of death (about 20%) caused by SIVs with no inflammatory residues on PB1-F2 in this co-infection model [15]. These experimental data support the possibility of SIVs expressing virulent variants of PB1-F2 proteins enhancing the development of secondary bacterial pneumonia in pigs and probably in other species. Interestingly, a recent publication by Buehler et al. [37] suggests that PB1-F2 from SIV is expressed at lower levels than those expressed by human IAVs and that, because of low expression levels, the SIV-derived protein may not have significant effect on virus pathogenicity. However, while there is a striking difference in expression in plasmid vectors, PB1-F2 expression in cells infected with intact SIV is relatively similar to those infected with human IAV [37]. Our data from above-mentioned studies with SIVs in a mouse model [15] are more consistent with the conclusion that either PB1-F2 is expressed reasonably well from intact SIVs, or that a low level of PB1-F2 expression is sufficient to support activity of virulent residues.

The substantial numbers of equine (73.8%), canine (22%), and swine (6.6%) IAVs with PB1-F2 proteins with the highest numbers (4 or 5) of virulent residues (inflammatory and cytotoxic combined) suggest a need for heightened and more thorough virologic surveillance among severe influenza cases (especially those complicated with secondary bacterial infection), combined with sequencing of PB1 and analysis of PB1-F2 proteins for the presence of virulence-associated amino acid residues in these animal hosts.

## Conclusions

Surveillance of novel potential virulence and transmission determinants other than in HA and NA is important for public health response and pandemic preparation. All H1N1, H2N2 and H3N2 pandemic viruses acquired PB1 segments from non-human viruses, demonstrating that this segment is capable of reassorting with human viruses and potentially altering pathogenicity of the resulting strains. Inflammatory and cytotoxic residues of PB1-F2 protein have been shown to increase IAV pathogenicity and the host's predisposition to secondary bacterial infection in a laboratory setting [13]–[15]. We previously determined that about 80% of avian H5N1 IAVs encode virulent (i.e., containing inflammatory and/or cytotoxic residues) PB1-F2 proteins [17]. From our current analysis, we can conclude that not only birds, but pigs, horses, and dogs are also reservoirs of pathogenic IAV PB1-F2 proteins.

We recognize that our conclusions are drawn from data with several limitations. Although the virulence of identified residues was indirectly confirmed in our numerous studies with PB1-F2 proteins from H1N1, H1N2, H2N2, H3N2, and H5N1 IAV lineages [11], [12], [15], [38]–[40], the verification of PB1-F2 residues virulence by direct mutagenesis was done only for H3N2 (inflammatory and cytotoxic residues; [13]) and H1N1 (cytotoxic residues; [14]) IAV lineages. In all cases the consequences for virulence were evaluated in mice only. We also do not know how intracellular localization of precise (full-length or truncated) PB1-F2 proteins may influence virulence of identified residues. Data from our studies do not indicate that inflammatory or cytotoxic residues realize their activity through mitochondrial targeting [13], [14]. However, we do not exclude that different sub-cellular localization of shortened or full length PB1-F2 proteins may influence activity of virulent residues. In addition, analyses of swine, equine, and canine PB1-F2 sequences for the presence of virulent residues were done with a non-random set of sequences present in public databases, suggesting the possibility of enrichment for more virulent IAV strains. Nevertheless, the presence of inflammatory or cytotoxic residues in swine, equine, and canine PB1-F2 proteins suggests a substantial potential for the enhancement of influenza severity in these animals and a need for surveillance of IAVs with genetic markers of virulence (i.e., inflammatory and cytotoxic residues of PB1-F2) that maybe emerging from these reservoirs. Further studies focusing on the role of PB1-F2 in the pathogenesis of swine, equine, and canine influenza will help to define the impact of the predicted virulence-associated amino acid residues.

## Supporting Information

Figure S1 **Phylogenetic analysis of the PB1 nucleotide sequences encoded by swine influenza A viruses.** Phylogenetic analysis for 789 H1N1, 529 H3N2, and 329 H1N2 SIVs are shown. Viruses with 1, 2, 3, and 4 inflammatory residues in PB1-F2 colored in blue, green, fuchsia, and red, respectively. Isolates with PB1-F2 truncated before residue 62 colored in cyan. Viruses with a cytotoxic residue are indicated by an asterisk. The amino acid combination at positions 62, 75, 79, and 82 (inflammatory) are shown on the right.
(PDF)

Figure S2 **Phylogenetic analysis of the PB1 nucleotide sequences encoded by equine and canine influenza A viruses.** Maximum likelihood phylogenetic trees for PB1 from 96 equine and 63 canine H3N8, 11 equine H7N7, and 19 canine H3N2 viruses were generated as described for Figure S1. Inflammatory residues of PB1-F2 colored in blue (1), green (2), fuchsia (3), and red (4). Cyan color indicates PB1-F2 protein truncated before residue 62. The presence of one or two cytotoxic residues is indicated by one or two asterisks, respectively. The amino acid combination at positions 62, 75, 79, and 82 (inflammatory) are shown on the right.
(PDF)

Table S1 We acknowledge the authors, originating and submitting laboratories of the sequences from GISAID's EpiFlu Database on which this research is based. The list is detailed below. All submitters of data may be contacted directly via the GISAID website www.gisaid.org.
(XLS)
