# Supplementary figures and images for: Non-Avian Animal Reservoirs Present a Source of Influenza A PB1-F2 Proteins with Novel Virulence-Enhancing Markers
Source: PLoS One. 2014 Nov 4;9(11):e111603. doi: 10.1371/journal.pone.0111603 (PMC4219726; doi:10.1371/journal.pone.0111603)

Swine H1N1 lineage

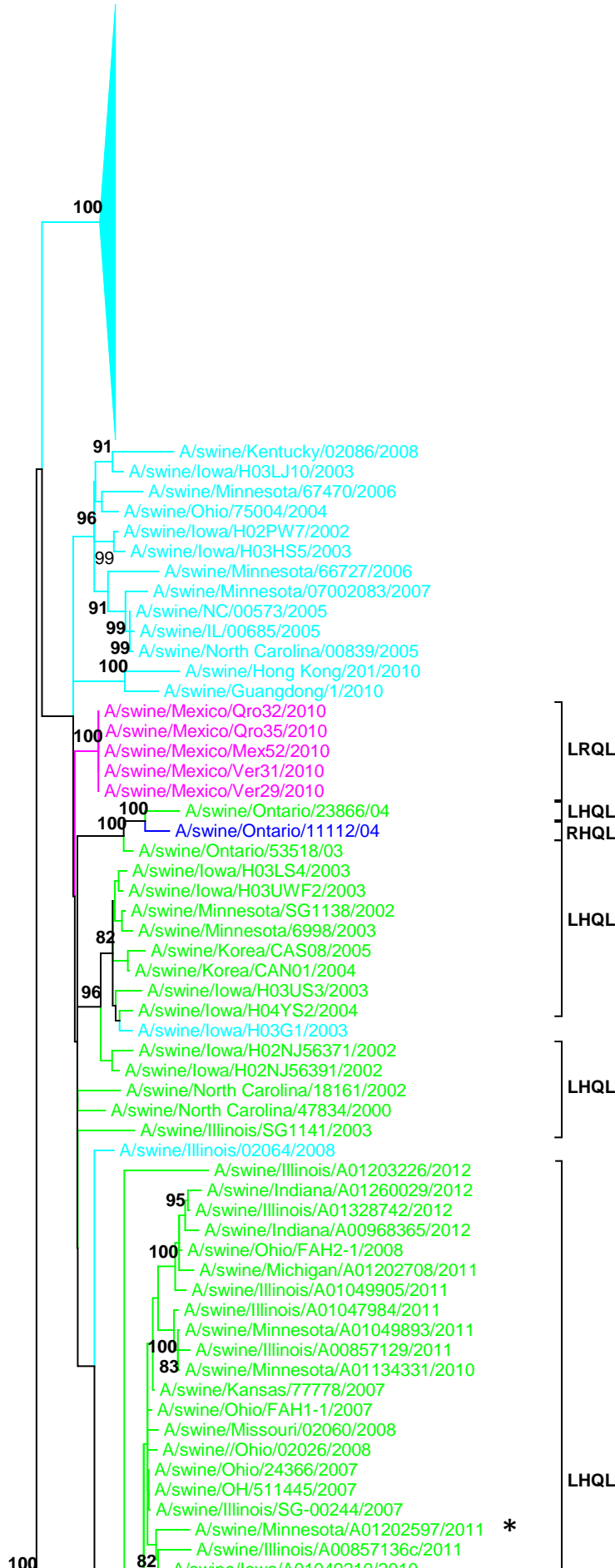

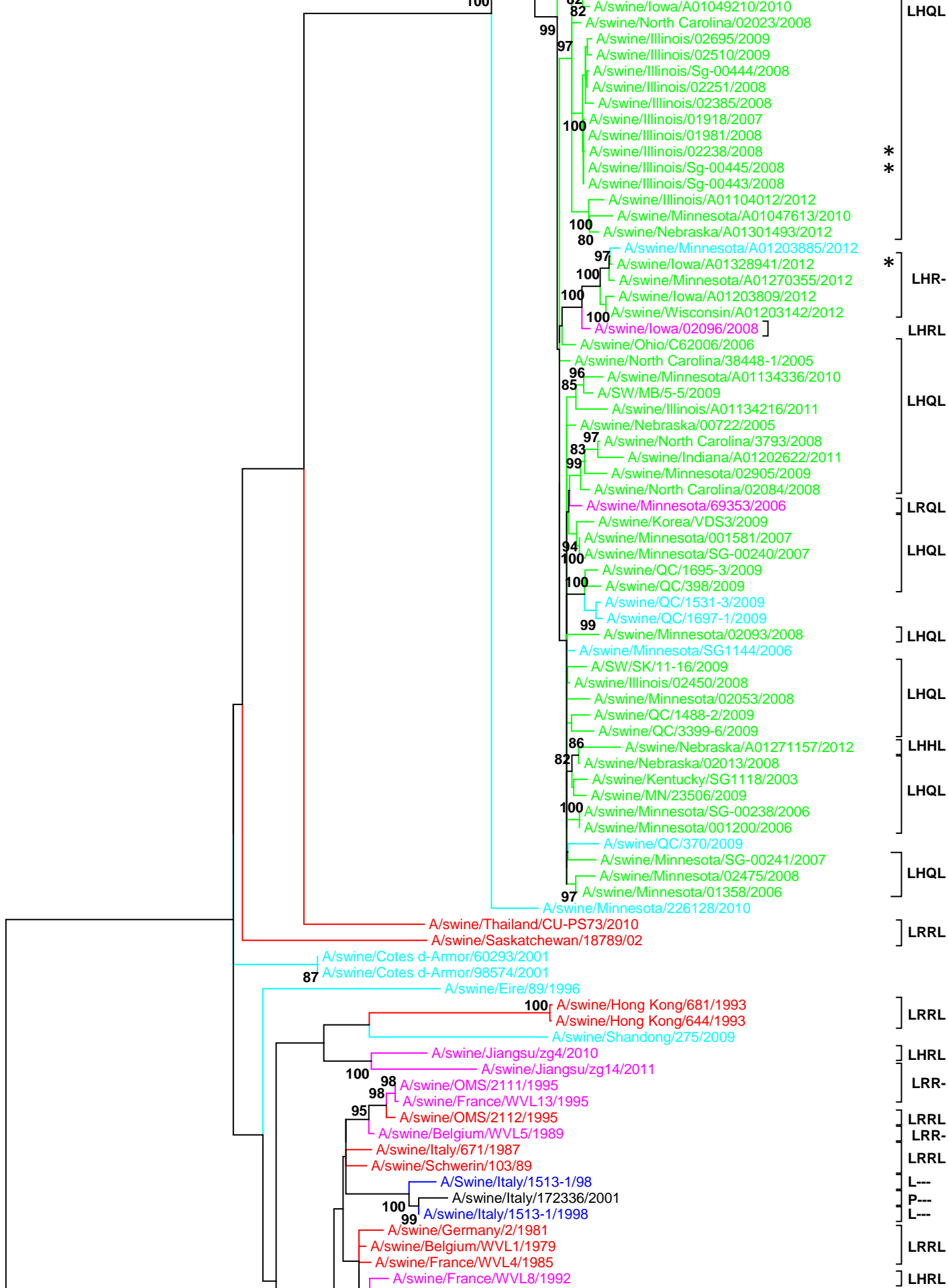

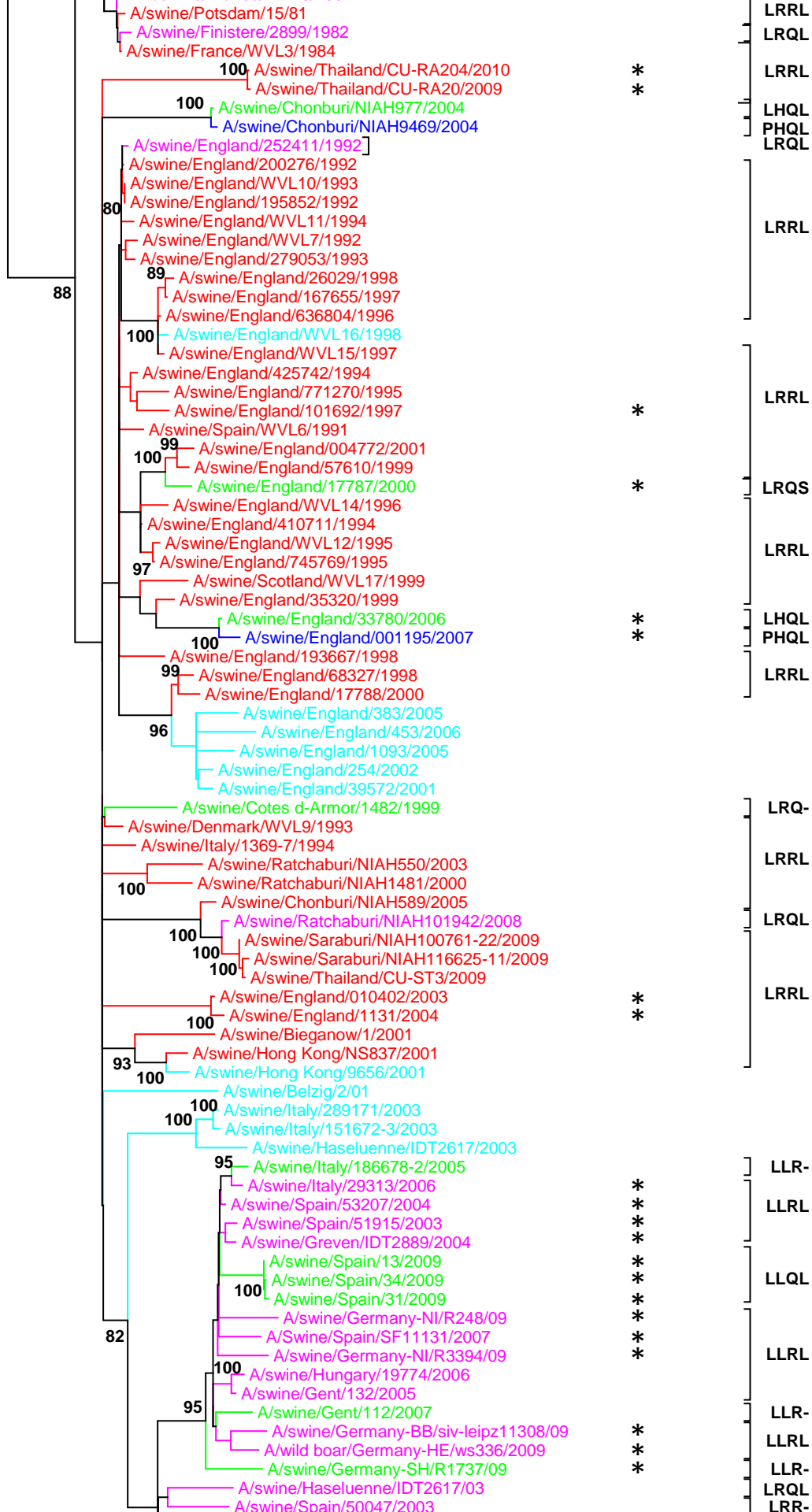

A/swine/Bakum/5/95

Spain/006/1999

100

0.05

# Swine H3N2 lineage

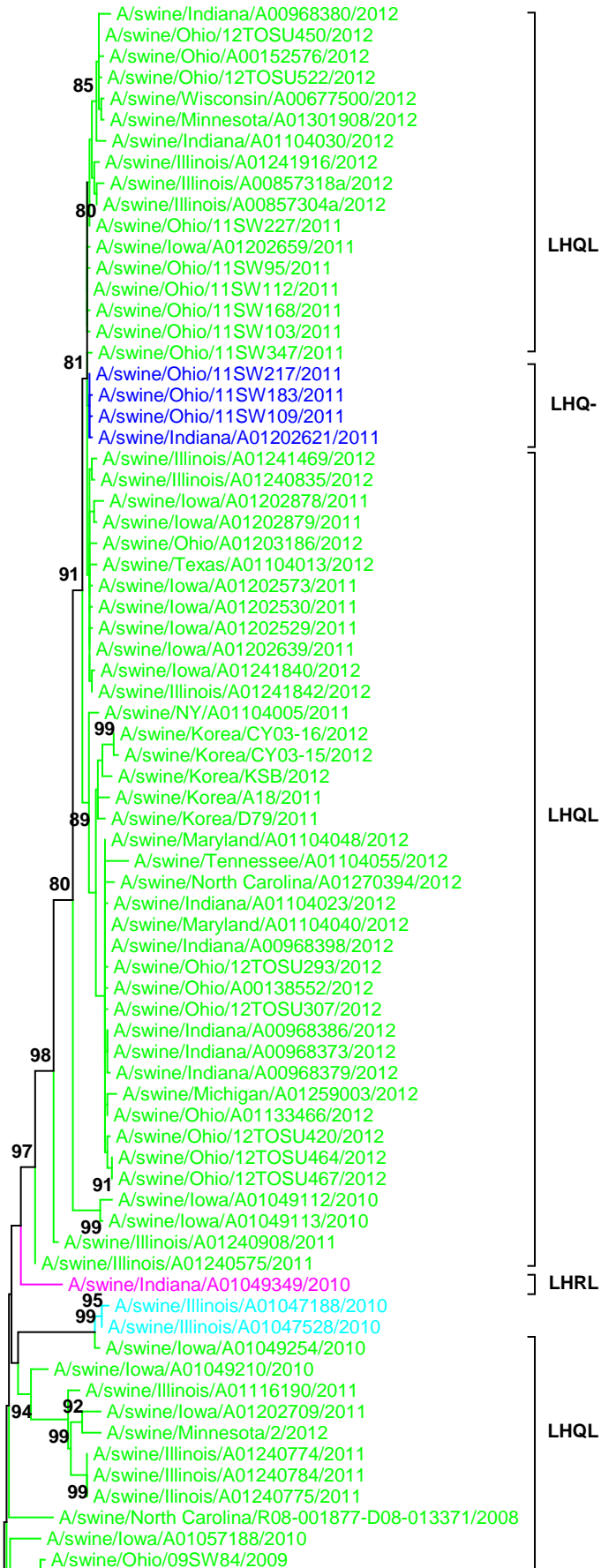

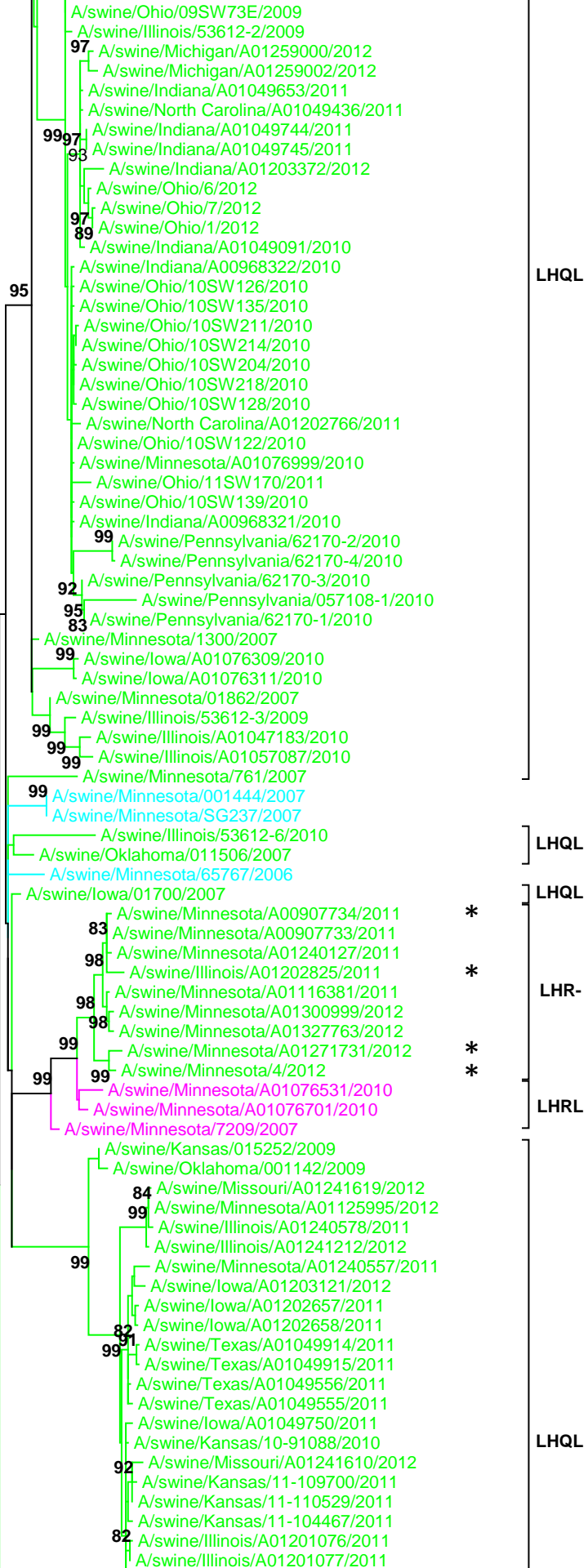

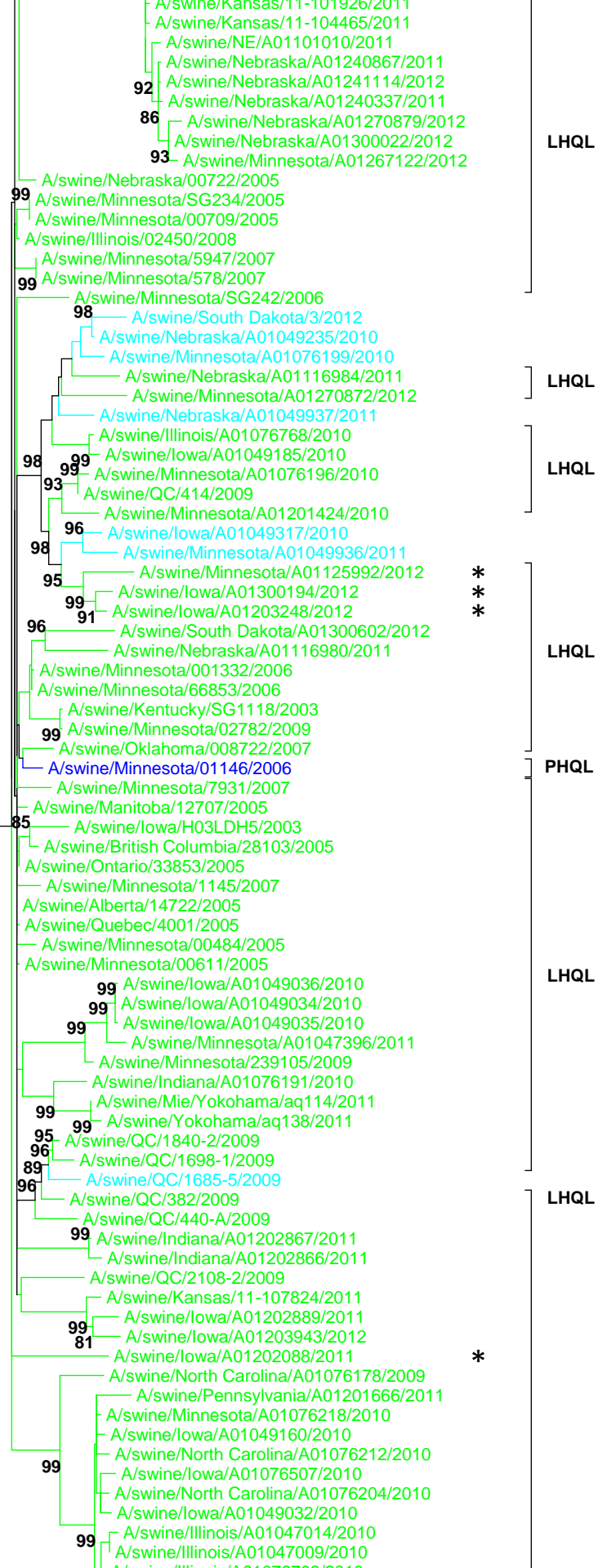

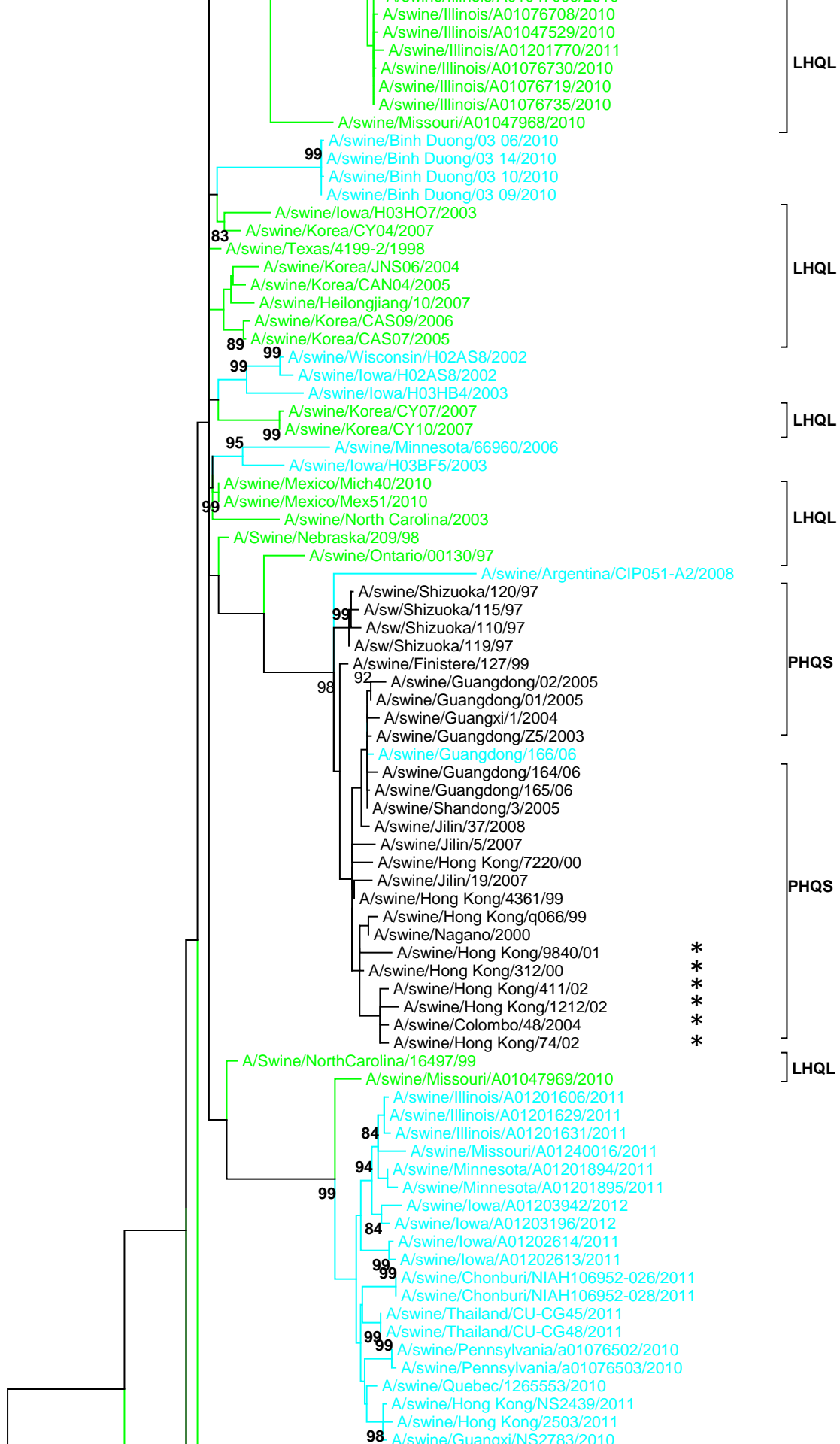

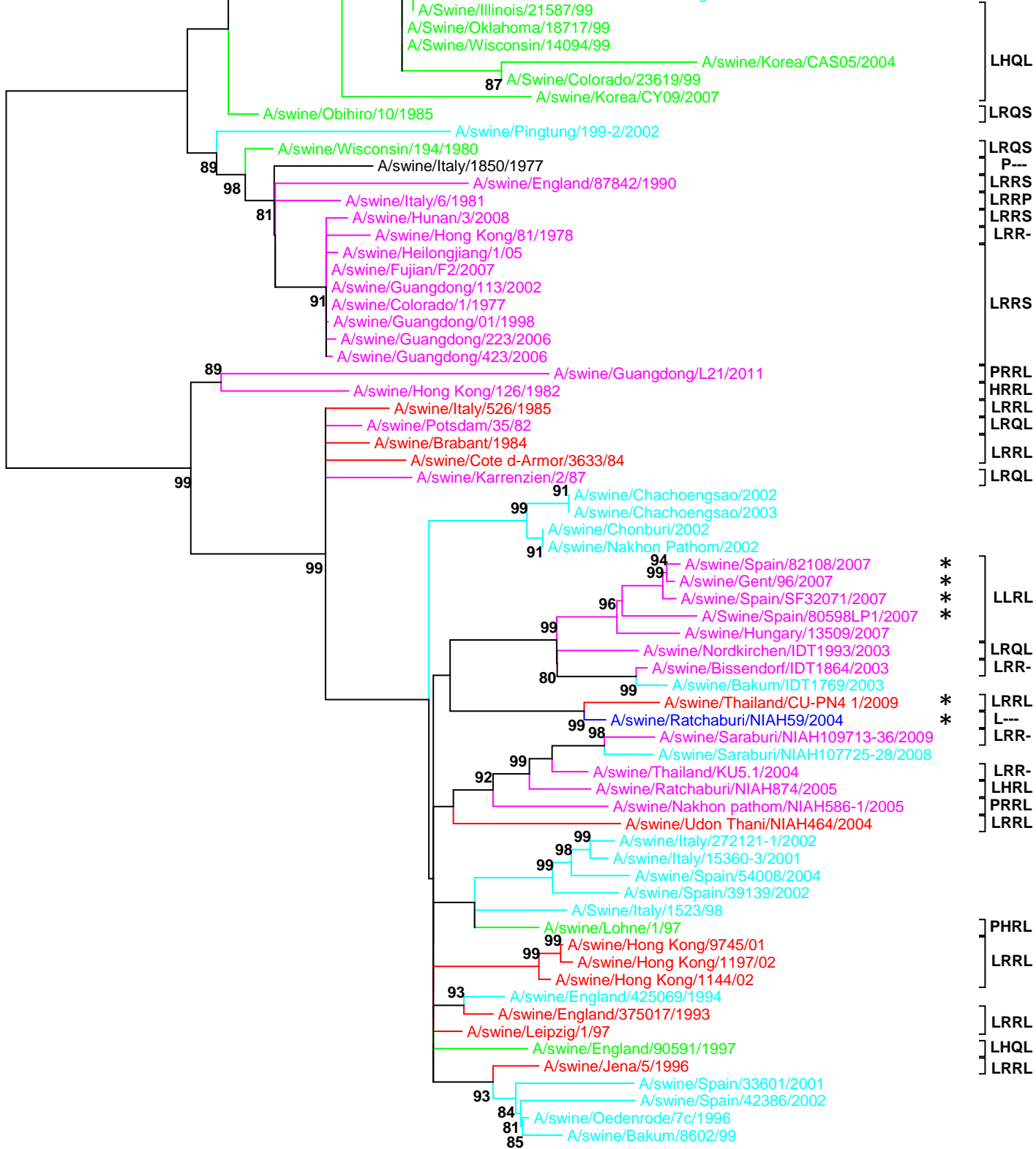

0.02

# Swine H1N2 lineage

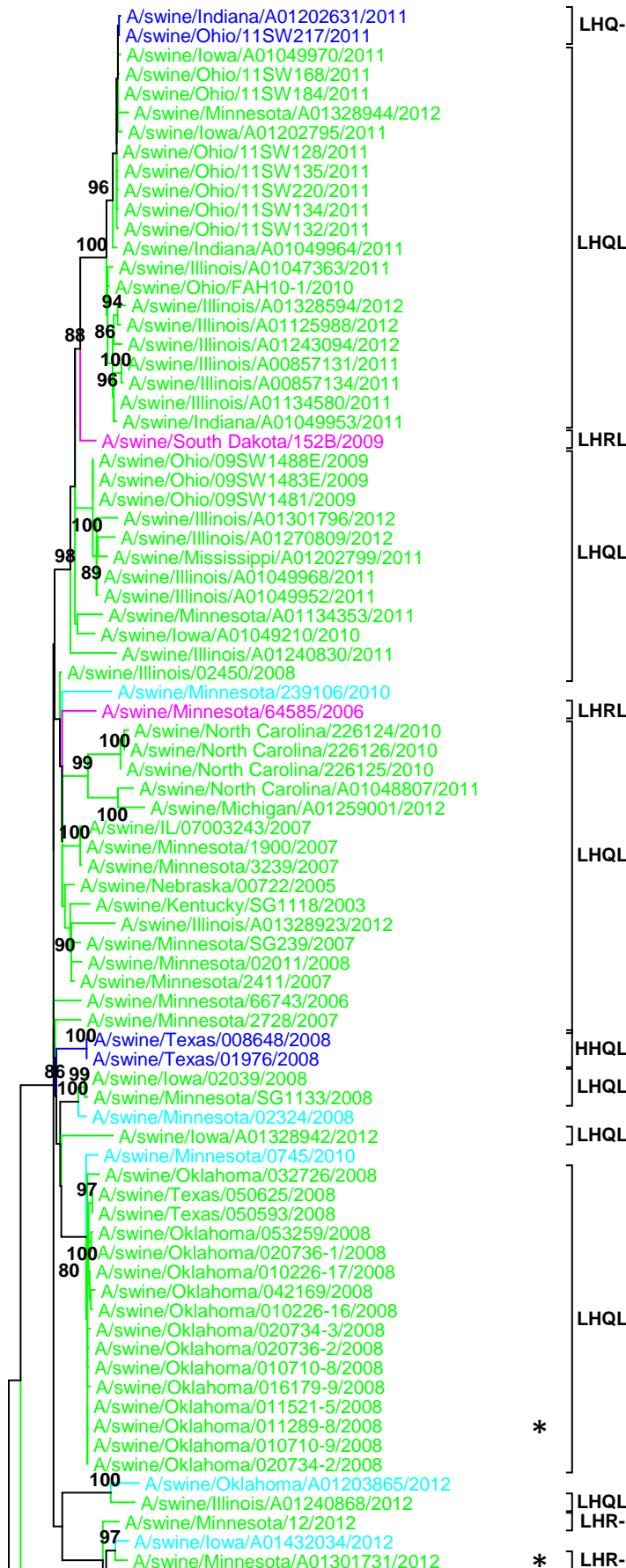

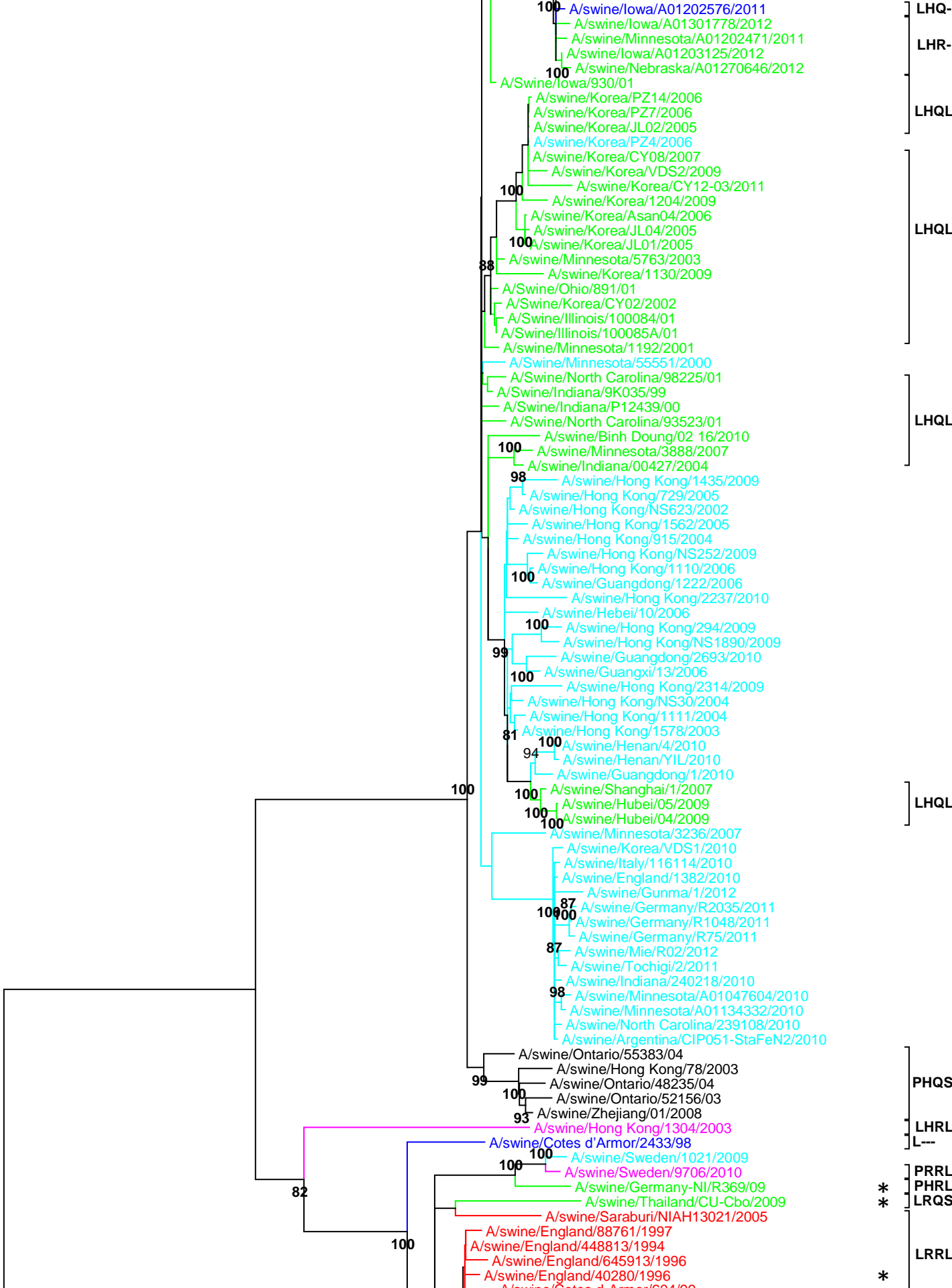

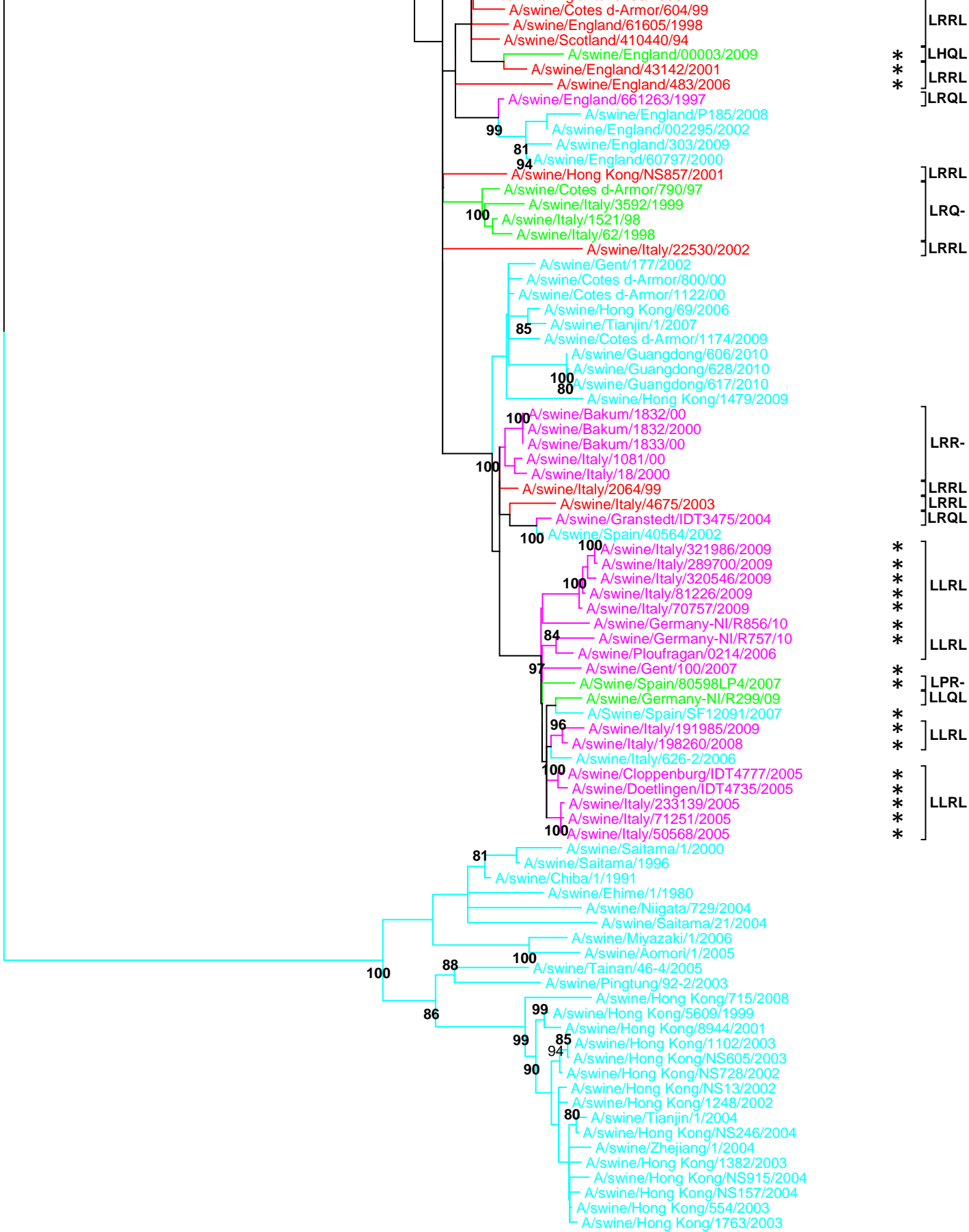

0.05

Supplement: Figure S1 — Phylogenetic analysis of the PB1 nucleotide sequences encoded by swine influenza A viruses. Phylogenetic analysis for 789 H1N1, 529 H3N2, and 329 H1N2 SIVs are shown. Viruses with 1, 2, 3, and 4 inflammatory residues in PB1-F2 colored in blue, green, fuchsia, and red, respectively. Isolates with PB1-F2 truncated before residue 62 colored in cyan. Viruses with a cytotoxic residue are indicated by an asterisk. The amino acid combination at positions 62, 75, 79, and 82 (inflammatory) are shown on the right. (PDF) [file pone.0111603.s001.pdf]

# Equine/Canine H3N8 lineage

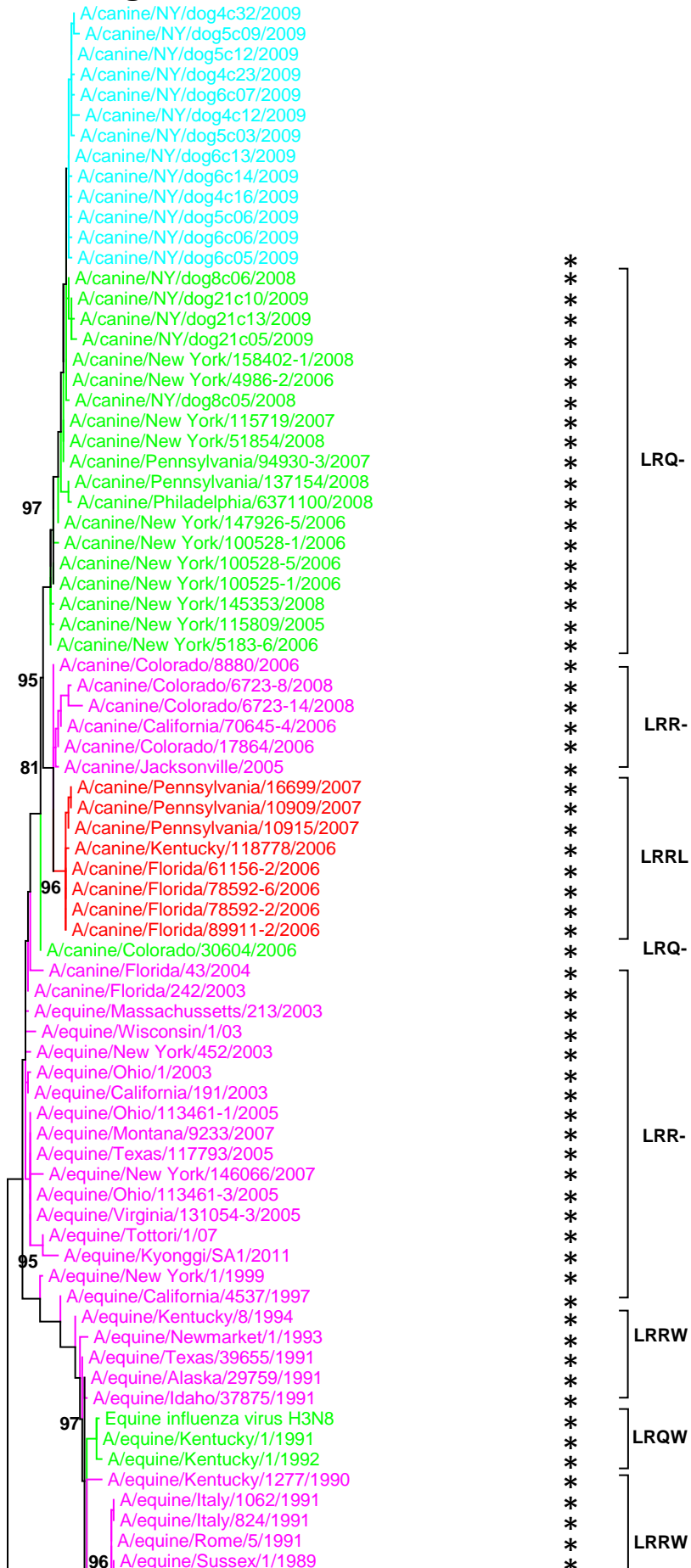

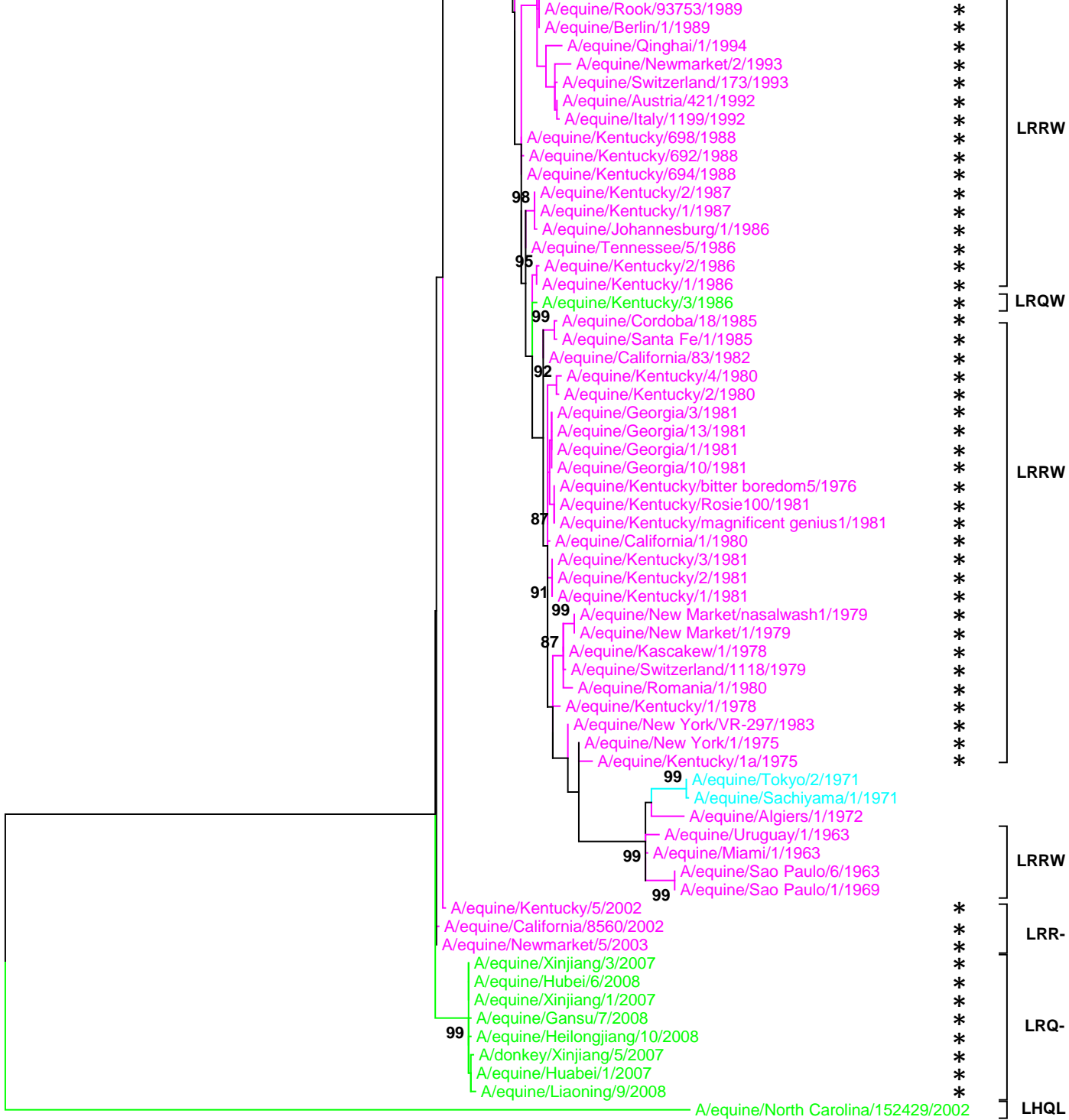

# Equine H7N7 lineage

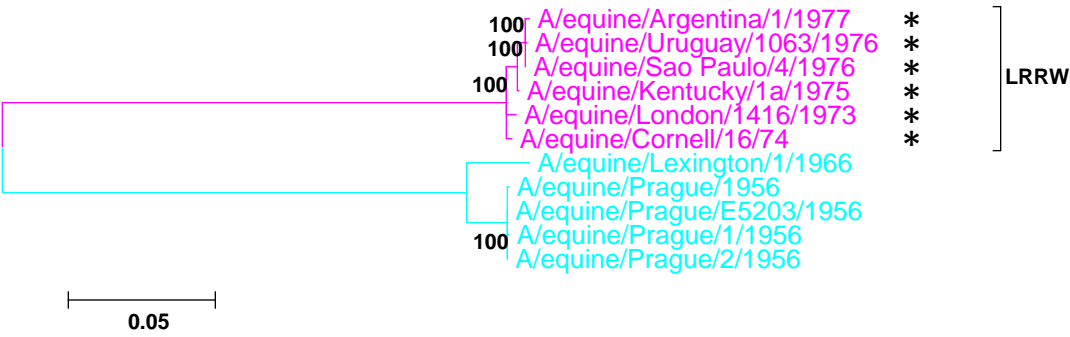

# Canine H3N2 lineage

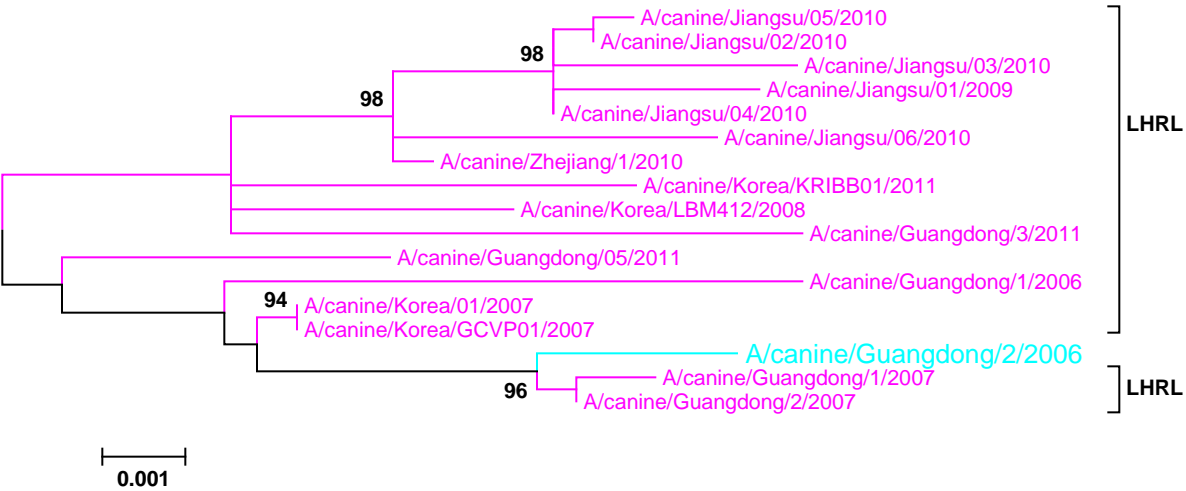

Supplement: Figure S2 — Phylogenetic analysis of the PB1 nucleotide sequences encoded by equine and canine influenza A viruses. Maximum likelihood phylogenetic trees for PB1 from 96 equine and 63 canine H3N8, 11 equine H7N7, and 19 canine H3N2 viruses were generated as described for Figure S1. Inflammatory residues of PB1-F2 colored in blue (1), green (2), fuchsia (3), and red (4). Cyan color indicates PB1-F2 protein truncated before residue 62. The presence of one or two cytotoxic residues is indicated by one or two asterisks, respectively. The amino acid combination at positions 62, 75, 79, and 82 (inflammatory) are shown on the right. (PDF) [file pone.0111603.s002.pdf]
